# Transarterial Radioembolization (TARE) Agents beyond ^90^Y-Microspheres

**DOI:** 10.1155/2018/1435302

**Published:** 2018-12-31

**Authors:** C. Bouvry, X. Palard, J. Edeline, V. Ardisson, P. Loyer, E. Garin, N. Lepareur

**Affiliations:** ^1^Comprehensive Cancer Centre Eugène Marquis, 35042 Rennes, France; ^2^Univ Rennes, CNRS, ISCR (Institut des Sciences Chimiques de Rennes), UMR 6226, 35000 Rennes, France; ^3^Univ Rennes, Inserm, LTSI (Laboratoire Traitement du Signal et de l'Image), UMR_S 1099, 35000 Rennes, France; ^4^Univ Rennes, Inra, Inserm, Institut NUMECAN (Nutrition, Métabolismes et Cancer), UMR_A 1341, UMR_S 1241, 35000 Rennes, France

## Abstract

Liver malignancies, either primary tumours (mainly hepatocellular carcinoma and cholangiocarcinoma) or secondary hepatic metastases, are a major cause of death, with an increasing incidence. Among them, hepatocellular carcinoma (HCC) presents with a dark prognosis because of underlying liver diseases and an often late diagnosis. A curative surgical treatment can therefore only be proposed in 20 to 30% of the patients. However, new treatment options for intermediate to advanced stages, such as internal radionuclide therapy, seem particularly attractive. Transarterial radioembolization (TARE), which consists in the use of intra-arterial injection of a radiolabelled embolising agent, has led to very promising results. TARE with ^90^Y-loaded microspheres is now becoming an established procedure to treat liver tumours, with two commercially available products (namely, SIR-Sphere® and TheraSphere®). However, this technology remains expensive and is thus not available everywhere. The aim of this review is to describe TARE alternative technologies currently developed and investigated in clinical trials, with special emphasis on HCC.

## 1. Introduction

Primary tumours (with hepatocellular carcinoma accounting for 75-80% of them) or secondary hepatic metastases are a major cause of death, with an increasing incidence [1, 2]. Hepatocellular carcinoma (HCC) is the fifth cancer in terms of incidence and the second leading cause of cancer death for men worldwide [3]. The vast majority of cases occurs in Southeast Asia [4], China alone representing half of the worldwide cases and deaths, and an increasing incidence in Europe. The presence of a cirrhosis, associated with various possible etiologies (such as hepatitis viral infections, alcohol, and haemochromatosis), is the primary risk factor. 73.4% of HCC can be attributed to hepatitis infections [5, 6], but the nature of the underlying disease mainly depends on geographical region or ethnic group. For instance, in Asia, 70-80% of all HCCs are attributable to hepatitis B virus (HBV) [4, 7], with the notable exception of Japan, where hepatitis C (HCV) is the main factor [4]. In the Western world, the main causes of HCC are alcoholism and nonalcoholic steatohepatitis (NASH), linked with a dramatic rise in obesity and diabetes occurrence, with also a strong increase in chronic hepatitis C [8, 9].

**Figure 1:**
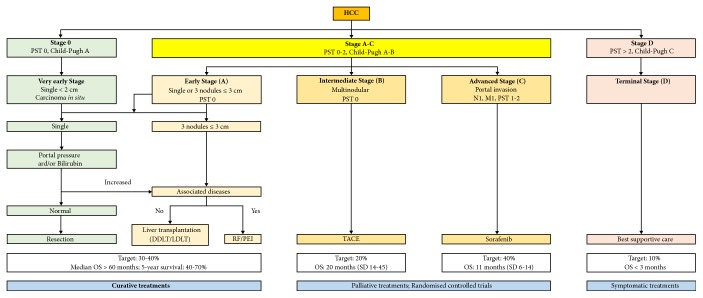
BCLC staging system and therapeutic strategy according to EASL-EORTC guidelines. © European Association for the Study of the Liver; European Organisation for Research and Treatment of Cancer. (Adapted from J Hepatol 2012; 56: 908-43.)

Despite the large range of treatment options developed over the years [10, 11], HCC prognosis remains dark, with a 5-year survival below 5%. The only really curative treatments are surgery (resection, transplantation) and ablative techniques (radiofrequency, cryotherapy, percutaneous alcohol injection). However, because of the underlying liver diseases (fibrosis, cirrhosis) and an often late diagnosis, a curative treatment can only be proposed in 20 to 30% of cases. Indeed, the presence of multiple foci, intraportal or extrahepatic metastases, and impaired hepatic functions are contraindications for liver transplantation or tumour resection. Additionally, even for such patients, recurrences are still likely to occur (up to 50% recurrence rate at 2 years for resection and, for transplantation, 10% within Milan criteria, but as high as 40% for patients outside Milan criteria) [12]. For nonoperable tumours, various palliative treatments may be proposed depending on the tumour staging. A large range of staging systems have been proposed [9, 13]. Most groups, especially in Europe, currently use the BCLC (Barcelona Clinic Liver Cancer), recommended by the EASL-EORTC (Figure 1). For intermediate stage HCCs, since external radiotherapy is of little use because of the high risk of hepatic toxicity, despite recent improvements in its efficacy and tolerance [14, 15], and systemic chemotherapy has not yet demonstrated effectiveness, with a low level of response without any improvement in survival [16], patients are generally proposed locoregional therapies [17]. For advanced HCCs, targeted treatment with sorafenib, and/or recently authorised regorafenib or other promising kinase inhibitors remains the only possibility [18, 19].

Due to both dual blood supply of the liver from the portal vein and the hepatic artery, and the sinusoidal cytoarchitecture of the liver parenchyma, the invasion of circulating tumour cells for establishing secondary hepatic metastatic foci is greatly favoured. 95% of hepatic malignancies are in fact secondary tumours [2]. So, for instance, liver metastases occur in approximately 50% of patients with colorectal carcinoma (CRC) [20], and in 44% of patients with neuroendocrine tumours (NET) [21]. Depending on volume and number of the metastases and histology of the original tumour, the median survival of patients with liver metastases ranges from 2 to 12 months [22]. To treat these intrahepatic metastases, surgery is considered the gold standard for CRC liver metastases [23] while its use remains controversial for metastases from non-colorectal primary tumours [21]. When surgery is not possible, because of the number of metastases, liver-directed therapies help in prolonging survival [17]. In the case of primary NET liver metastases, peptide-receptor radionuclide therapy (PRRT) with radiolabelled somatostatin analogues seems appealing [24–26].

Taking advantage of this dual blood supply and a rich vasculature, intra-arterially delivered treatments, such as transarterial chemoembolization (TACE) and transarterial radioembolization (TARE), appear as really attractive treatment modalities. TACE, consisting in intra-arterial injection of an emulsion of Lipiodol and a chemotherapeutic drug, followed by the occlusion of the feeding artery, is currently considered as the standard of care for intermediate stage HCCs and some metastases [27], but its effectiveness remains a matter of debate. In this context, the development of a well-tolerated treatment modality appears particularly attractive. TARE, consisting in the intra-arterial delivery of a radioactive material to the tumour, limiting systemic irradiation and preserving the healthy liver to a maximum extent, appears to be a promising alternative to TACE [28–31]. Different materials (Lipiodol, glass, resin, or polymer microspheres) and radioisotopes have been used [32]. Only a few radionuclides have suitable characteristics for the treatment of tumours, among them are ^32^P, ^90^Y, ^131^I, ^166^Ho, ^177^Lu, and ^186/188^Re, all of which are *β*-emitters (Table 1). ^90^Y-loaded microspheres now have an established role and proved to be safe and effective in treating primary and secondary liver cancers, with favourable toxicity profile, with several tens of prospective or retrospective clinical studies completed or ongoing [15, 33–35]. There are, however, few Phase III studies, and published results are up to now disappointing [36, 37], but mostly because of suboptimal study designs or inadequate patient selection. Moreover, though this treatment modality has gained wide acceptance, it remains very expensive, especially for low-income countries, and is not available everywhere. Though not as common as TARE with ^90^Y-microspheres, TARE alternative methods are under clinical investigation, either in primary or in secondary liver cancers and will be presented here.

## 2. TARE with Radiolabelled Microspheres

The idea to utilise radiolabelled microspheres to treat tumours, liver being the first organ successfully treated, dates back to the 1960s [38]. First isotopes used were phosphorus-32 (E_*β*max_ = 1.71 MeV; t_1/2_ = 14.3 d, max tissue penetration = 8 mm) and yttrium-90 (E_*β*max_ = 2.28 MeV; t_1/2_ = 64.0 h, max tissue penetration = 12 mm), two pure *β*-emitters. It then fell out of use, before being revived in the 1980s until present day. There are currently two commercially available ^90^Y-labelled microspheres, based on different technologies [39]. One is based on glass microspheres (TheraSphere®, BTG International Ltd., London, United Kingdom); the other is made of ion-exchange resin (SIR-Sphere®, SIRTEX Medical Limited, North Sidney, New South Wales, Australia). Another TARE device, loaded with holmium-166 (E_*β*max_ = 1.84 MeV; t_1/2_ = 26.8 h, max tissue penetration = 8.7 mm), has been recently made available (QuiremSpheres®, Quirem Medical BV, Utrecht, Netherlands) and is currently the subject of clinical trials in liver metastases. Besides these, numerous types of microspheres with various sizes and labelled with different isotopes have been developed and studied in humans [40].

### 2.1. Phosphorus-32 Microspheres

Phosphorus-32 was among the first isotopes used for therapeutic purposes, thanks to its suitable properties. In the middle of the 1960s, Caldarola et al. [41] used ^32^P-labelled resin microspheres for intra-arterial treatment of tumours. In 1979, Grady used ^32^P-CrPO_4_ colloids to treat intrahepatic metastases in a pilot study [38]. During the time of follow-up (2 years), 3 out of 4 patients were doing well, without significant side effects. However, with the rise of ^90^Y, ^32^P fell out of use [42], except in China where 44 patients with unresectable liver cancer were treated with moderate results with ^32^P-glass microspheres [43] and in Iran where 39 patients suffering from primary or secondary liver cancer were recently treated with intra-arterial injection of ^32^P-CrPO_4_ colloids [44]. This study was, however, based on imaging parameters and no result on the outcome of the patients is given.

### 2.2. Holmium-166 Microspheres

Compared with ^90^Y, ^166^Ho has the advantage of possessing a *γ* emission (81 keV) suitable for SPECT imaging. Moreover, holmium is highly paramagnetic, thus enabling MRI imaging and quantification [45, 46]. It has a 26.8 h half-life, resulting in a high dose-rate, and can be produced pure upon neutron bombardment, since natural holmium-165 has a 100% abundance and a large cross section [47]. ^166^Ho-loaded microspheres have been prepared using synthetic polymers [48], natural polymers [49], phosphate [50], resin [51], or glass [52].

#### 2.2.1. Glass Microspheres

Glass is relatively resistant to radiation damage and nontoxic. Glass microspheres can easily be spheroidized in uniform sizes and can generally be produced with minimal radionuclidic impurities [53]. Holmium doped (Ho_2_O_3_) aluminium silicate glasses were “melted in an alumina crucible at 1600°C in a crucible type electric furnace. The liquid was held at this temperature for 2 hours and stirred each 30 min by using a silica rod to assure the liquid homogenization and gas release”. Brown et al. [54] prepared ^166^Ho-loaded glass particles with a small size of 2-5 *μ*m for direct injection into mammary carcinoma tumours in mice, which led to an effective deposition of intense *β*-radiation. It could therefore be an effective modality for use in localised internal radionuclide therapy. Yet, no further studies were reported. In 2009, Costa et al. reported the preparation of 20-50 *μ*m diameter glass particles (considered to be the ideal particle size to reach the arterioles of the liver), loaded with a specific activity up to 224 GBq/g of microsphere, suitable for therapeutic applications [52]. Their reported microspheres however contained more than 20% of ^177^Lu, because of the presence of lutetium oxide in the initial material.

#### 2.2.2. Resin Microspheres

Compared with glass microspheres, ion-exchange resin-based microspheres present with the advantage of a lower density and commercial availability. Schubiger investigated different resins with ^90^Y and found that Bio-Rex 70 (Bio-Rad Inc, Hercules CA, USA) was the most suitable one [55]. Bio-Rex resin is made from acrylic polymer, with carboxylic groups used to bind the radiometal. Subramanian et al. recently investigated the feasibility of using Bio-Rex 70 with ^166^Ho [56]. ^166^Ho-labeled microspheres were obtained in high-yield (94.53% at pH 8.5), demonstrated high* in vivo* stability and showed very good retention in the liver (94.94 ± 1.51% at 72 h pi.). Besides Bio-Rex resins, Aminex resins (Bio-Rad Inc, Hercules CA, USA) were also explored. Aminex A-5, based on styrene divinylbenzene copolymer with sulphonic acid functional groups, was used [51]. Biodistribution in pigs demonstrated a reproducible fixation in the liver and dosimetry study, based on SPECT, allowed determining the required radiation absorbed dose to the liver to be 25 Gy to reach a therapeutic activity.

#### 2.2.3. Polymer Microspheres

**Figure 2:**
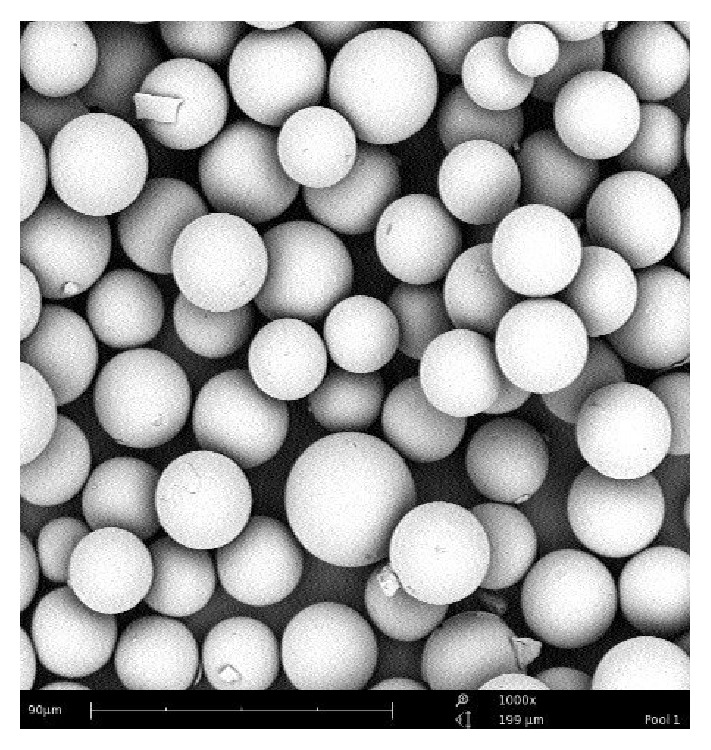
Scanning-electron microscope image of Holmium-PLA microspheres (from [73]).

Polymer-based microspheres present with the advantage of near-plasma density, biodegradability, and biocompatibility [48]. However, this material is not able to withstand high thermal neutron fluxes [57, 58]. To circumvent this problem, microspheres can be loaded with holmium after neutron irradiation, like Suzuki et al. with chitosan microspheres [59] or Zielhuis et al. with alginate microspheres [49]. An alternative is to use additives and to select carefully the irradiation parameters (like avoiding any traces of water, < 1h irradiation in a high-flux reactor) [47, 60]. Another important parameter is the stability of the holmium-166 inside the polymer matrix. Mumper et al. showed that *β*-diketone chelates and particularly acetylacetonate (AcAc) formed strong complexes with holmium and that it moreover had a strong melting point amenable to withstand the heat inside the reactor without melting [61]. He thus developed poly-(L-lactic acid) (PLA or sometimes PLLA) microspheres for the prospective treatment of liver tumours, prepared by a simple solvent evaporation technique [62]. This method was soon used and furthered by a team from Utrecht [60]. Briefly, [^165^Ho]-AcAc complex and PLA are dissolved in chloroform, a volatile and water immiscible solvent, and subsequently added to polyvinyl alcohol aqueous solution. The resulting solution is emulsified by stirring, thus forming small organic droplets until the organic solvent evaporates. As chloroform evaporates, the droplets harden, forming microspheres, which can be collected through filtration. Microsphere size can be tuned through speed of stirring. The resulting microspheres, after drying, are then irradiated into a nuclear reactor to yield the desired ^166^Ho-loaded microspheres. Chloroform is a toxic solvent and should be avoided in patients. It has however been demonstrated that irradiation resulted in the complete removal of chloroform from the microspheres [63]. Holmium-loaded PLA-microspheres produced by this method (Figure 2) have been extensively characterised [47, 58, 64, 65] and are now produced under Good Manufacturing Practices [66]. Biodistribution studies demonstrated an effective tumour targeting in rats, with a 6.1 ± 2.9 tumour-to-liver ratio at one day after administration [67]. Animal studies have also established a low toxicity profile in rats followed up to 18 months, with only slight chronic inflammation, and no release of holmium load, as assessed with MRI [68]. They also demonstrated that correct administration of the microspheres was a critical step [69] and that it was feasible to quantify the dose through MR imaging [70, 71] and to predict dosimetry with the use of a scout dose of ^166^Ho-PLLA-microspheres [72].

A phase I trial for the treatment of unresectable, chemorefractory, liver metastases was consequently initiated [73]. 15 patients were treated with escalating aimed whole-liver absorbed doses of 20, 40, 60, and 80 Gy. At 6 weeks, treatment response was 1 partial response (PR), 7 stabilised disease (SD), and 7 disease progression (DP). At 12 weeks, treatment response was 1 PR, 1 SD, and 13 DP. The only PR was obtained for a patient treated with 20 Gy to the liver. Results from this trial showed “^166^Ho-radioembolization is feasible and safe for the treatment of patients with liver metastases” and enables image-guided treatment [74]. Maximum tolerated dose was determined to be 60 Gy. MRI and SPECT-based dosimetry were compared and found to be equivalent, with overall T/N ratios ranging from 0.9 to 2.7 with SPECT and 1.1 to 3.1 with MRI [75]. Use of a scout dose ^166^Ho-microspheres to assess lung shunting and predict dosimetry was demonstrated to be more accurate than ^99m^Tc-MAA imaging [76] and proven to be safe, as possible extrahepatic deposition of the microspheres was estimated to have a low incidence of 1.3% [77]. Based on these promising results, a phase II study was launched, with a fixed aimed whole-liver dose of 60 Gy (or 3.8 GBq/kg of liver tissue, including the scout dose), in patients with liver metastases refractory to systemic therapy and ineligible for surgical resection (38 patients were included, one of whom was not evaluable). Besides ^166^Ho-scout dose to calculate dosimetry, a scout dose of ^99m^Tc-MAA was administered to assess the lung shunts prior to treatment. In the recently published results [78], the target lesions showed complete response, partial response, or stabilised disease at 3 months for 27 patients (95% confidence interval [CI], 57% – 85%). The median overall survival was 14.5 months (95% CI, 8.6 – 22.8 months). The toxicity profile was acceptable. Compared to ^90^Y-microspheres TARE, the presence of *γ*-emission does increase radiation exposure to some extent, but only patients treated with more than 7 GBq of ^166^Ho and released within 6 h after treatment (instead of 24 h) would require contact restrictions. In all other cases, patients can be released without contact restrictions [79]. This study thus demonstrated clinical efficacy and further studies should be undergone. Another clinical study is currently ongoing, comparing a newly developed anti-reflux catheter to standard microcatheter for radioembolization of patients with liver-dominant colorectal metastases [80].

In parallel to the development of ^166^Ho-PLLA-microspheres for liver metastases, ^166^Ho-chitosan microspheres were investigated for HCC treatment [59]. Chitosan is a chitin derivative which can chelate metals and solidifies at alkaline pH. A phase IIb trial evaluated percutaneous injection of this device in 40 patients with small HCC [81], while another phase II trial investigated transarterial injection in 54 patients with a single large HCC [82]. Both studies reported good results, with survival rates at 1, 2, and 3 years of 87.2%, 71.8%, and 65.3%, respectively, in the first study and tumour necrosis achieved in 77.5% of patients with HCC < 3cm and 91.7% of patients with HCC < 2 cm. In the second study, where tumour size ranged from 3 to 13 cm, response rate was 78%, with 31 patients having a complete response for a 27-month median duration. Two treatment-related deaths were reported. Authors nonetheless found the toxicity to be acceptable, but patients should be carefully selected. When categorising patients according to their tumour size, it was found that patients with tumours of an intermediate size (3–5 cm) were the optimal candidates for this treatment. Phases III trials are therefore justified.

### 2.3. Rhenium-188 Microspheres

Rhenium-188 (E_*β*max_ = 2.12 MeV; E*γ* = 155 keV (15%); t_1/2_ = 16.9 h, max tissue penetration = 11 mm), available through a ^188^W/^188^Re generator, offers cost-effective convenient on-site availability, short half-life, energetic *β*-particle, and emission of *γ*-photons suitable for imaging [83, 84]. It is thus a promising isotope for radionuclide therapy, especially treatment of liver malignancies. Various particle materials have been considered for ^188^Re-microspheres preparation [85], but, so far, only albumin microspheres have been investigated in human.

#### 2.3.1. Glass Microspheres

Chemically durable yttrium alumino–silicate (YAS) microspheres, incorporating more than 50 wt% of rhenium oxide (ReO_2_), have been prepared and neutron-irradiated in high-flux nuclear reactor [86]. These microspheres were investigated in hepatoma bearing rats, following intra-arterial injection, where they proved to be safe and effective in diminishing tumour growth [87]. However, low-cost and highly available natural rhenium consists of two neutron activatable radioisotopes (^185^Re and ^187^Re, both having large cross-sections for neutrons, yielding therapeutic amounts within a few h), thus producing a mixture of ^186^Re and ^188^Re, both *β*- and *γ*-emitting radionuclides, with different energies and different half-lives (cf. Table 1). The major disadvantage is that dosimetric calculations are complicated because of the two radioisotopes that must be considered. The alternative would be to use enriched ^187^Re, but at a much higher cost.

#### 2.3.2. Resin Microspheres

There is only one example in the literature of resin-based microspheres loaded with ^188^Re [88]. Aminex A-27 resin (Bio-Rad Inc, Hercules CA, USA) was labelled by “adding [^188^Re]-perrhenate and SnCl_2_ to vacuum-dried resin particles. The mixture was boiled and centrifuged and microspheres were separated and resuspended in saline”. They were tested in hepatoma-bearing rats by direct intra-tumoural injection. “Survival over 60 days was significantly better in the treated versus control group (80% versus 27%)”. The same team compared this method with percutaneous ethanol injection in VX2 rabbit model [89]. Mean survival was 38.8 ± 6.2 days for the control group (rabbits treated with saline injection), 55.8 ± 11.8 days for the percutaneous ethanol injection group, and 68 ± 9.8 days for the rabbits treated with ^188^Re-microspheres. They thus concluded intra-tumoural injection of ^188^Re-microspheres could be an alternative to percutaneous ethanol injection and to intra-arterial injection.

#### 2.3.3. Polymer Microspheres

Diverse natural and synthetic polymers have been tested to prepare ^188^Re-microspheres [85]. Poly(L-lactic acid) was mixed with jet-milled rhenium powder and the microspheres were prepared with the solvent evaporation similar to the method described above with holmium. 22 *μ*m microspheres were neutron-activated for 3 h to give ^188^Re-PLA-microspheres [90]. Unfortunately, these biodegradable microspheres were damaged by the high neutron fluxes needed to achieve sufficiently high specific activity required for the treatment of liver tumours. This strategy was therefore abandoned. An alternative way to load PLA microspheres is to encapsulate a ^188^Re-labelled chelate inside the polymeric microspheres. Shukla et al. [91] encapsulated ^188^Re-DMSA, with a low 20-30% yield, while Yu et al. [92] derivatised PLA with a poly(histidine) tag to attach the [^188^Re(CO)_3_(H_2_O)_3_]^+^ complex directly onto the polymer chains. They thus reported a radiolabelling efficiency of 92%. Recently, encapsulation of ^188^Re-sulfur colloids in PLA microspheres by an oil in‐water (O/W) emulsion solvent extraction procedure was reported [93]. The 13-48 *μ*m particles were labelled with over 99% efficiency and were injected intravenously into mice, showing high lung uptake. ^188^Re-colloids were also encapsulated in poly(L-lactide-co-glycolide) (PLGA) microparticles, along with doxorubicin, and used for transarterial chemoradioembolization in F344 rats [94]. ^188^Re/DOX@MS showed stronger tumour inhibition than ^188^Re@MS and DOX@MS alone.

Biodegradable starch microspheres have been proposed as an embolization agent to protect healthy liver tissue during TARE with ^90^Y-microspheres [95]. Verger et al. [96] proposed to chemically modify starch so that it could chelate ^188^Re and developed a cold-kit labelling method to prepare ^188^Re-starch-based microspheres with > 95% labelling yield. When injected in DENA-induced rats, the activity was essentially located in the tumorous parts of the liver. In the same way, human serum albumin (HSA) labelled with technetium-99m has been used for years for lung perfusion imaging, and ^99m^Tc-labelled macroaggregates of albumin (MAA) are the current method of choice to assess lung shunts and predict ^90^Y-microspheres dosimetry [97]. In 2000, Wunderlich et al. proposed to label HSA with ^188^Re [98]. They found that HSA B20 (ROTOP Pharmaka GmbH, Dresden, Germany) gave uniform microparticles with a mean 25 *μ*m diameter and were easily and stably labelled with generator-produced ^188^ReO_4_Na in the presence of tin chloride and HSA. They also demonstrated high* in vivo* stability, with a preferential lung uptake when injected intravenously in rats. A cold kit method was subsequently developed [99]. The proposed kit, consisting of three vials, enabled an almost quantitative labelling yield and minimum handling. The preparation was recently further optimised by Chen et al., using microwave heating, thus shortening the reaction time from 1 h to 3 min [100]. Tumour volume in the normal saline treated group was 1803.2 mm^3^ at 54 days after tumour inoculation, while tumour volumes in the 103.6 MBq and 240.5 MBq of ^188^Re-HSA microspheres treated groups were 381 and 267.4 mm^3^ (P = 0.001 and 0.004), respectively, demonstrating the high therapeutic efficacy of this modality.

^188^Re-HSA microspheres have been investigated in a feasibility study in 10 patients with HCC (3) or colorectal liver metastases (7) [101]. Patients were given0 13.6 ± 4.7 GBq of ^188^Re-microspheres selectively in the feeding artery of the tumour. The urinary excretion rate was low (8.9 ± 3.8%IA within 96 h). Response was assessed on CT: 2 patients had PR, 5 patients had SD, and 3 patients had DP. The treatment was well tolerated, with an acceptable rate of toxicity (30% of grades I and II, and 10% of grade III toxicity), which was reversible in most patients within 14 days after treatment. This treatment could thus represent a therapeutic option in these patients, but it is not possible to draw any firm conclusions, because of the small size and heterogeneity of the patient group. A larger patient cohort is necessary. A second study using ^188^Re-HSA microspheres was described by Nowicki et al. [102]. This study included 13 patients with progressive primary or secondary liver tumours. They received 3.8–12.4 GBq. The urinary excretion rate was low (6.5 ± 2.3%IA within 48 h). Mean overall survival was 7.1 months, and progression free survival was 5.1 months. Treatment adverse events were at an acceptable level, with 4 patients having grade 3 toxicity, mostly due to cancer progression. This study confirms the results of the first study and ^188^Re-HSA microspheres deserve to be further investigated.

## 3. TARE with radiolabelled Lipiodol

The most used vector for intrahepatic administration is Lipiodol. Lipiodol is an ethyl ester of iodinated fatty acids derived from poppy seeds with a proportion of iodine of approximately 38% by weight (i.e., 475 mg/mL). This iodised oil was discovered in 1901 by Guerbet and is the first organic iodinated contrast agent used for X-ray, in lymphography. Nakakuma et al. have shown that Lipiodol is selectively captured by hepatoma and by certain hepatic metastases, of colonic, neuroendocrine and mammary origin [103]. Lipiodol has therefore been used for the detection of HCC, then also for vectorising chemotherapeutic substances. Intratumour retention time has also been shown to be significantly greater than retention time in healthy liver, with retention in tumour cells up to several months [104]. Replacement of cold iodine with iodine-131, by an exchange reaction on Lipiodol [105], makes it possible to obtain radiolabelled Lipiodol, ^131^I-Lipiodol, which was successfully used for the treatment of nonoperable HCC [106, 107]. Still, Iodine-131 possesses a high energy gamma emission and long half-life (E_*β*max_ = 0.81 MeV; E*γ* = 364 keV (81%); t_1/2_ = 8.02 d) requiring hospitalisation in a radioprotected room for several days, thus limiting its potential. Other therapeutic nuclides, such as Yttrium-90 [108] and Rhenium-188 [109], have been proposed to advantageously replace it. Radiolabelling and clinical application of Lipiodol with Rhenium-188 have been extensively reviewed elsewhere [110].

### 3.1. Lipiodol Radiolabelling

**Figure 3:**
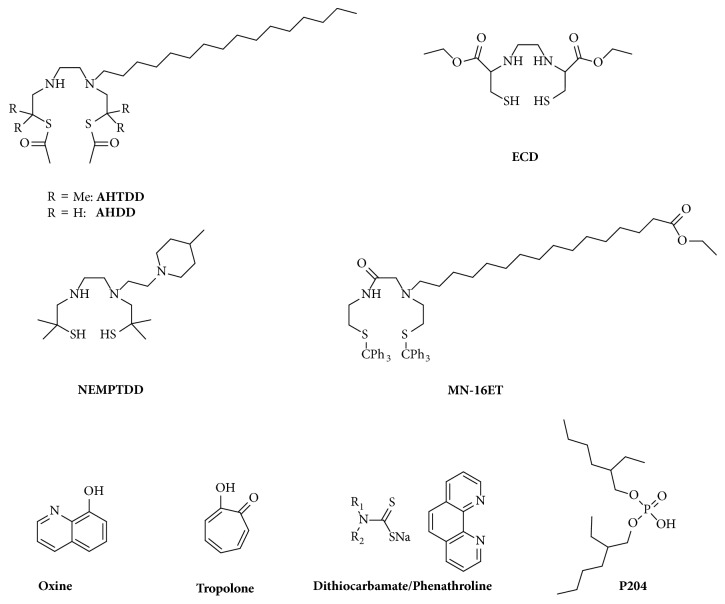
Ligands investigated with radiometals (^188^Re and ^90^Y/^166^Ho/^177^Lu) for Lipiodol labelling.

**Figure 4:**
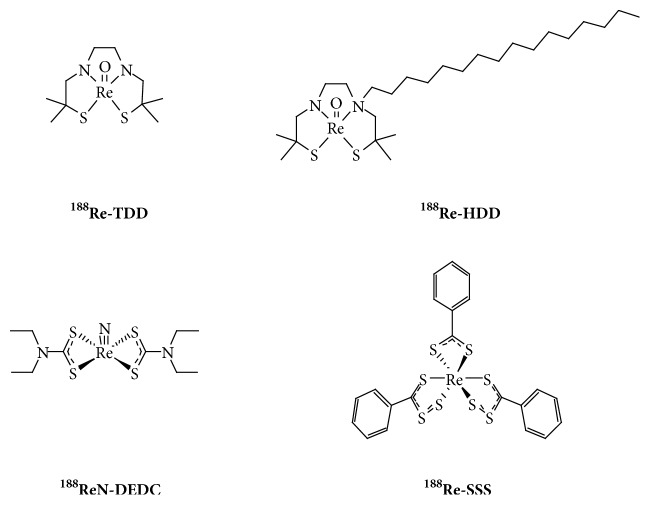
^188^Re-chelates used to label Lipiodol and evaluated in human.

Initial trials to covalently label Lipiodol with ^188^Re and ^90^Y revealed unsuccessful [111–113]. Solubilisation of a lipophilic radiocomplex into Lipiodol was suggested as more suitable [114]. Several such ^188^Re-labelled complexes [115–127] were subsequently proposed (Figures 3 and 4).

Lipiodol was also labelled with lipophilic chelates of yttrium-90 and radiolanthanides, although literature is scarce compared with rhenium-188. Oxine (8-hydroxyquinoline) forms a lipid-soluble complex with trivalent metals and has been used since the 1970s with indium-111 to label leukocytes [128]. Based on this, it has been proposed to label Lipiodol with yttrium-90 [129], holmium-166 [130], and lutetium-177 [131]. The procedure was however rather time-consuming and the resulting complex lacked sufficient stability, leading to undesirable bone uptake. Using the same analogy with ^111^In-labelled leukocytes, ^90^Y-tropolonate has been investigated to label Lipiodol [132]. The radiotracer showed increased stability compared to oxinate and high tumour uptake, but its stability was not satisfactory enough. Other compounds mentioned in the literature include dithiocarbamate/phenanthroline ^90^Y-complex [133] and di(2-ethylhexyl) orthophosphoric acid (P204), initially developed for solvent extraction of metals [134], with no biological data. Ligands used are summarised in Figure 3.

Since handling of high activities of beta-emitting therapeutic radionuclides can lead to a high radiation burden to the staff performing the preparations [135, 136], remote-controlled procedures were developed [137–139]. This led to a significant reduction of the received dose [140], in addition to a more GMP-compliant preparation.

### 3.2. Clinical Outcome

^131^I-labelled Lipiodol (Lipiocis®) dates back to the 1980s [141, 142]. Since then, many feasibility studies and a few Phase III trials have been carried out, essentially for the management of inoperable HCCs [107, 110, 143]. Three small randomised controlled trials were carried out for the palliative treatment of HCC with portal vein thrombosis (PVT) and without PVT, and adjuvant treatment after surgery, with respectively 129, 27, and 43 patients. Despite encouraging results, because of the suboptimal properties of ^131^I and a more than expected lung toxicity [144], Lipiocis® is not commercially available anymore in Europe. In-house labelled ^131^I-Lipiodol is however still under use in some countries, like for instance India [139]. To date, besides ^131^I-Lipiodol, only ^188^Re-labelled Lipiodol has been assessed in human. No ^90^Y- or radiolanthanide-labelled Lipiodol has been thus far investigated in clinics. Four ^188^Re-chelates have been evaluated to prepare clinical ^188^Re-labelled Lipiodol [110], i.e., ^188^Re-TDD [115], ^188^Re-HDD (now rebadged HTDD) [117], ^188^ReN-DEDC [125], and ^188^Re-SSS [126] (Figure 4). Except one nonconclusive case report with ^188^Re-TDD/Lipiodol [145], and two dose-escalation studies with ^188^ReN-DEDC/Lipiodol [125] and ^188^Re-SSS/Lipiodol [146], which both demonstrated promising preliminary outcomes, most clinical studies were carried out with ^188^Re-HDD/Lipiodol [147–158]. Despite high urinary excretion [150] and difficulties to obtain high activities because of low labelling yields [118], ^188^Re-HDD/Lipiodol demonstrated favourable responses and appeared to be well tolerated in dose escalation trials [148, 149], and in various feasibility studies in more advanced forms of the disease [151, 152], as well as in second-line therapy after relapse following a curative treatment [153, 154].

Following these encouraging results, an international IAEA-sponsored multicentric Phase II trial was launched [156–158]. 185 patients were included, receiving 1 to 4 treatment doses, with activities ranging from 0.78 to 13.45 GBq. The outcome of this study was 25% objective response (with 3% CR), 53% stabilised disease and 22% tumour progression, with a median follow-up of 455 days. One- and two-year survivals were, respectively, 46 and 23%. Tolerance was good. In view of these results, a larger randomised Phase III study appears to be required.

#### 3.2.1. Other Therapeutic Applications

Lipiocis® had received marketing authorisation for the treatment of HCC with PVT. ^131^I-Lipiodol was however also investigated in other indications, such as in adjuvant and neoadjuvant settings, where it, respectively, led to an increase in overall and disease-free survivals [159] and to a decreased risk of recurrence before transplantation or resection [160, 161]. To date, only one preliminary study (with 5 patients) used ^188^Re-Lipiodol to stabilise patients awaiting liver transplantation, demonstrating its feasibility [162]. The advantage of ^188^Re-Lipiodol, compared to ^131^I-Lipiodol, is the shorter half-life of ^188^Re, making patients eligible for transplantation after 1 week, while they had to wait for 4 weeks when treated with ^131^I-Lipiodol. More patients are needed to be able to conclude on its potential use in this indication.

Besides HCC, liver can be home to other primary and secondary tumours, for which TACE with Lipiodol has shown its efficiency and tolerability [163, 164]. Limited studies using TARE with ^131^I-lipiodol have been published though. Contradictory results have been obtained in pilot studies with cholangiocarcinoma (CCA) [165, 166] and TARE is thus not recommended [167], while treatment of hepatic metastases could prove to be valuable [168, 169]. It would therefore be interesting to assess the potential interest of ^188^Re-Lipiodol in these indications, for which radiolabelled microspheres (with either ^90^Y, ^166^Ho, or ^188^Re) have proven their safety and efficacy.

## 4. Conclusions

Research on treatments for primary or secondary liver tumours has been particularly active, at the preclinical and clinical levels, following the successful introduction of ^90^Y-microspheres in the therapeutic armamentarium. Among the many methods to be investigated, ^188^Re-Lipiodol appears as a relatively economical, safe, and attractive alternative to ^90^Y-microspheres for the treatment of HCC. ^166^Ho-microspheres also look particularly appealing, thanks to the multimodal properties of holmium-166, and initial clinical findings, especially for liver metastases. Large randomised Phase III trials now need to take place, to confirm these encouraging Phase I/II results. In order to be able to define the place of TARE in treatment decision plans, comparisons with other existing treatment modalities, notably targeted therapies and newly developed immunotherapies, must be undertaken. It is most probable that future treatment strategies may rely on the combination with one or another of these therapies. Ultimately, choice of the TARE modality will be made based on local expertise and availability. Besides, many aspects of TARE still remain a challenge, such as optimisation of specific activity, determination of embolic distribution, microdosimetry, and personalised dosimetry (pre- and post-injection), as well as predictive response factors, requiring further clinical and preclinical studies.

## Figures and Tables

**Table 1:** Radionuclides used for TARE.

**Radionuclide**	**t**_**1/2**_ ** (days)**	**E**_β_ ** (MeV) (**%**)**	**E**_γ_ ** (keV) (**%**)**	**Tissue penetration range (mm)**	**Production method**
^32^P	14.3	1.71 (100)	/	7.9	Nuclear reactor

^90^Y	2.7	2.284 (100)	/	12	^90^Sr/^90^Y generator
					Nuclear reactor for microspheres labelling

^131^I	8	0.81 (90)	0.364 (81)	2	Nuclear reactor

^166^Ho	1.1	1.84 (50.5)	81 (6.4)	8.7	Nuclear reactor

^177^Lu	6.7	0.497 (79)	113 (6.4)	2.2	Nuclear ractor
			208 (11)		

^186^Re	3.8	1.07 (72)	137 (9)	4.5	Nuclear reactor

^188^Re	0.7	2.118 (72)	155 (15)	11	^188^W/^188^Re generator
					Nuclear reactor

Note: t_1/2_ (days), radioisotope half-life in days; E_*β*_ (MeV) (%), maximum particle energy and respective decay abundance shown in parentheses; E_*γ*_ (KeV) (%), gamma ray energy useful for imaging and respective abundance in total energy emission shown in parentheses; tissue penetration range (mm), maximum tissue penetration shown in millimeters.
